# Motivational Interviewing and Self-Care in Type 1 Diabetes: A Randomized Controlled Clinical Trial Study Protocol

**DOI:** 10.3389/fendo.2020.574312

**Published:** 2020-12-10

**Authors:** Dácil Alvarado-Martel, Mauro Boronat, María del Pino Alberiche-Ruano, María Andrea Algara-González, Yolanda Ramallo-Fariña, Ana M. Wägner

**Affiliations:** ^1^ Endocrinology and Nutrition Department, University Hospital Insular Materno-Infantil, Las Palmas de Gran Canaria, Spain; ^2^ Research Institute of Biomedical and Health Sciences (IUIBS), University of Las Palmas de Gran Canaria (ULPGC), Las Palmas de Gran Canaria, Spain; ^3^ Evaluation Unit (SESCS), Canary Islands Health Research Institute Foundation (FIISC), Tenerife, Spain; ^4^ Research Network on Health Services in Chronic Diseases (REDISSEC), Madrid, Spain

**Keywords:** type diabetes 1, adherence, self-care, motivational interviewing, self-efficacy, health related quality of life, randomised controlled trial

## Abstract

**Background:**

Type 1 diabetes is a disease with complex therapeutic recommendations that require day-to-day lifestyle changes. Motivational Interviewing is a communication tool that has proved effective in changing behaviors in people with addictions, obesity and type 2 diabetes. Our objective is to evaluate the effects of a Motivational Interviewing intervention in people with type 1 diabetes.

**Methods:**

Sixty-six patients with type 1 diabetes and hemoglobin A1c >= 8% have been included and randomly assigned (computer-generated sequence, sealed envelopes, ratio 1:1) either to the intervention or to the control group. In the intervention group, appointments every 4 months with the endocrinologist include Motivational Interviewing; in the control group, the appointments proceed as usual. Patients will be followed for 16 months. The primary outcome will be self-care behaviors, assessed by a validated questionnaire, the Diabetes Self-Care Inventory-Revised Version. Secondary outcomes include: HbA1c, motivation for self-care, self-efficacy, health-related quality of life, satisfaction with professional-patient relationship, and fulfillment of patients’ own objectives. The practitioners receive training in Motivational Interviewing in order to help them promote adherence to self-care, encourage patient motivation and improve the doctor-patient relationship. The Motivational Interviewing intervention will be evaluated by two psychologists, blinded to the assigned treatment, through video recordings of the sessions and the administration of a purpose-built questionnaire, the EVEM 2.0 scale.

**Discussion:**

There is evidence that MI can improve self-care in type 2 diabetes. In this study, we aim to evaluate the effect of MI on self-care and HbA1c in people with type 1 diabetes.

**Clinical Trial Registration:**

https://clinicaltrials.gov/ct2/show/NCT03906786, identifier NCT03906786.

## Highlights

- Research has shown that the level of motivation reported by the patient was the strongest predictor of adherence to self-care, and was also recognized as one of the greatest obstacles to adherence to treatment.
- Motivational Interviewing has shown to be effective in changing behaviors in people with addictions, obesity, and type 2 diabetes.
- The present trial will assess the effects of Motivational Interviewing on adults with type 1 diabetes.

## Background

People with type 1 diabetes (T1D) have to carry out a series of complex tasks every day in order to manage their disease and maintain good glycemic control (1). These tasks include glucose measurements, multiple insulin injections, carbohydrate counts in the diet, and management of hypoglycemia and hyperglycemia. In addition, patients must continuously make decisions regarding the adjustment of their insulin treatment and the everyday problems related to their illness (2–4). In a previous study which identified multiple factors related to self-care, the level of motivation reported by the patient was the strongest predictor of adherence to self-care, and was also recognized as one of the greatest obstacles to adherence to treatment (5). Currently, diabetes management is focused on person-centered care and empowerment, a philosophy that encourages patient involvement, awareness and participation in decisions concerning their health (6). One of the fundamental pillars for promoting empowerment of people in their self-care is therapeutic patient education (7, 8). Therapeutic patient education provides patients with the knowledge, tools and skills needed to facilitate informed decision-making and self-management of the disease (8). However, therapeutic education alone does not guarantee the involvement of patients in the care of their health (8, 9).

Since 2014, in its *Standards* for *Diabetes* Self-Management *Education and Support*, the American Diabetes Association recommends a set of evidence-based communication strategies which have been shown to facilitate behavioral changes and to complement therapeutic patient education. They include cognitive strategies, problem solving, enhanced self-efficacy, relapse prevention strategies and Motivational Interviewing (MI) (10, 11). To quote the creators of MI: “Motivational Interviewing is a collaborative, goal-oriented method of communication with particular attention to the language of change. It is designed to strengthen an individual’s motivation for and movement toward a specific goal by eliciting and exploring the person’s own argument for change within an atmosphere of acceptance and compassion” (12).

MI emerged for the first time in Norway in 1982, and initially focused on addictions (13). Since then, its use has spread to other areas. It has been widely applied in the management of behavior-associated diseases (14) and more than three decades of research have established it as an effective approach for improving a series of health-related behaviors (14). Currently, there is moderate, consistent and robust evidence that MI achieves behavioral changes and improves adherence to treatment in patients with alcohol abuse, unhealthy lifestyles (e.g., sedentarism, overeating) and obesity/overweight (15, 16). Brief 15-min MI interventions have also proven to be effective, though the probability of success increases with the number of encounters and with longer follow-up periods (14).

MI has been shown to be effective in improving glycemic control in people with type 2 diabetes (T2D) (16–20) and its superiority over cognitive behavioral therapy has also been demonstrated (21). MI has proved effective in reducing HbA1c in adolescents with T1D (22, 23), and in a clinical trial it also improved quality of life (24, 25). It is effective as a complement to therapeutic education (26) and to other treatments, or as an independent treatment (22). However, not all studies have reported improvement in glycemic control in T1D or T2D (27, 28). A recent systematic review, which included four randomized controlled trials, concluded that there is some evidence of benefit of MI in people with T1D, but that more research is needed to isolate the effect of MI alone on adherence to treatment and HbA1c (29).

### Objective and Hypothesis

The purpose of this randomized controlled clinical trial is to study the impact of the application of MI in routine follow-up visits of patients with T1D and poor metabolic control.

The hypothesis is that an intervention with MI can increase patients’ self-care behaviors, reduce HbA1c, enhance their self-efficacy and health-related quality of life, and improve the doctor-patient relationship.

## Methods

This study protocol was developed in accordance with the Recommendations for Interventional Trials 2013 Statement (SPIRIT 2013) and the Consolidated Standards of Reporting Trials statement (CONSORT 2010) when applicable.

### Theoretical Framework

MI is defined primarily by its spirit: that is, a style that nurtures the interpersonal relationship between the therapist and the patient. It explains how the process of change is formed by different stages and how people are more likely to abandon a habit at certain stages than at others. In addition, it considers that deciding to change, to commit oneself, and to take responsibility for a therapeutic process is key to achieving behavior changes. MI uses a style of collaborative communication focused on one or several objectives and pays special attention to the language of change. It is designed to strengthen personal motivation and commitment to a specific goal, eliciting, and exploring the reasons that the person has for changing, in an atmosphere of acceptance and empathy. The approach is based on patient-centered counseling, cognitive therapy, systems theory and the social psychology of persuasion. It integrates clinical skills that promote motivation, combining managerial and non-managerial elements. In sum, it is a practical and specific contribution to daily life in which language is used to influence behavior by helping individuals express their own internal motivations, and guiding conversations in such a way that people persuade themselves to change in accordance with their own values and interests.

For maximum benefit to be derived from MI, the interviewer must have received adequate training, but must also believe in it in order to be able to influence the patient and initiate a process of change. MI involves eliciting from the patient what they already know and have, rather than giving them what they lack (for instance, knowledge, or medication) (30). This focus can improve patients’ motivation, since they choose what they want to do or change, not what others want them to do or change. By developing MI skills, practitioners can help patients identify what matters to them and then use these motivations to bring about changes in their health behavior (30).

Assessing the effectiveness of MI in the context of diabetes may appear controversial, since adherence to self-care already obliges people with diabetes to carry out multiple tasks. In fact, previous studies have focused on assessing the impact of MI on glycemic control (14, 17); to date, there are no clinical trials that study its impact on adherence to self-care behaviors in adults with T1D, or evaluate the standardized application of MI by practitioners.

### Study Design and Setting

This randomized, controlled, single-blind, parallel group clinical trial is carried out at the Endocrinology and Nutrition service of the Insular University Hospital, Las Palmas de Gran Canaria, a reference center in its area for the care of people with T1D.

### Participants and Recruitment

A total of 66 people with T1D have been included in the study and will be followed over a period of 16 months. Patients who met the inclusion criteria were informed of the nature of the study and invited to participate. Subsequently, they were recruited by their endocrinologist at the outpatient clinics of the Endocrinology and Nutrition Service of the Insular University Hospital. The recruitment period lasted from March to August 2019.

### Inclusion Criteria

Diagnosis of T1D, age over 18, at least one year of disease duration, HbA1c >= 8% and/or severe hypoglycemia in the previous 6 months.

### Exclusion Criteria

Pregnancy either in progress or scheduled in the following 12 months; any other circumstance that, in the opinion of the investigators, might interfere with the follow-up.

Patients who meet the inclusion criteria receive written information regarding the study and are invited to participate. They are also provided with more information by phone, when they are reminded that the trial will begin at the next scheduled follow-up appointment. At this first appointment, prior to the consultation, the participants sign the informed consent document and fill in a dossier that records all the clinical, sociodemographic and psychosocial variables. These variables are listed in the Measures section below.

### Randomization and Blinding

The participants are randomly assigned to the intervention group or control group. A computer-generated randomization list (with a 1:1 ratio) is used for each endocrinologist to prepare the labels indicating the assignment of each patient, which is kept in sealed envelopes, numbered consecutively, and stored at the unit. At the time of inclusion of a patient in the study (appointment 1), the appropriate envelope is chosen and opened to show the endocrinologist the treatment assigned. The randomization is stratified by practitioner and the treatment assigned to the first participant each day is applied to all participants scheduled on that day, in order to facilitate the application of the MI and avoid contamination between treatment groups. To limit possible bias, patients are not informed of the assigned treatment. The informed consent document explains that the study will evaluate the effects of the doctor-patient communication, though without going into detail. The researcher who generated the randomization list and prepared the envelopes for the assignment of the treatment group will not be involved in the treatment or evaluation of the participants; likewise, the researchers who will rate MI compliance and analyze the data will be blind to the treatment allocation.

### Intervention

The intervention will consist in the application of MI by the endocrinologist at four follow-up visits, held at 4-month intervals.

These visits proceed in the same way as standard appointments, with the addition of this new clinical approach. The visits last approximately 15–20 min, in accordance with routine hospital practices.

At each visit, the practitioner applies the four processes of MI (12):

- Engaging. This first stage serves to establish a therapeutic relationship of trust and mutual respect, if this has not already been achieved.
- Focusing. This stage is a continuous process of searching for and maintaining direction. The aim is to place the emphasis on the “focus”, i.e., the aspect that the person wants to change. It is not a static process; it may be that in other stages it is necessary to change course and renegotiate the objectives.
- Evoking. This stage is aimed at eliciting “change talk” from the patient, so that he or she persuades him or herself to change. It begins with the expression of a desire, reason, or need. This stage promotes self-efficacy, one of the most powerful predictors of successful behavior. MI promotes change because it impacts on people’s self-efficacy.
- Planning. This stage is characterized by the presence of specific actions. There is less maintenance talk (regarding the behavior to be changed) and more change talk. The person begins to visualize the change and imagines the possible positive results. MI ends when the person commits to the plan.

Before the first appointment and 4 months after the fourth appointment, the participants complete the questionnaires. In each of the five visits the HbA1c value will be recorded.

A sample of 25 baseline appointments 1 was videotaped in order to assess the application of MI, and serve as ongoing training for the practitioners. A similar procedure will be followed for appointment 4. The practitioners who apply MI have previously received training.

#### Professional Training

MI is a set of skills designed to help patients overcome their ambivalence to change by evoking motivation and commitment. Professionals can gain expert experience in this method, for example, through an eight-step program (Motivational Interviewing Network of Trainers- MINT), which can be completed in 16 h.

The three endocrinologists have received structured MI training through theoretical-practical workshops taught by a psychologist with previous training and experience in MI. The workshops, which included didactic instruction and interactive exercises, were divided into two 3- to 4-h sessions and two to four additional 60- to 90-min encounters. The training started 3 months before recruitment, continued until the intervention began and addressed the contents established by MINT (**Table 1**). Interactive exercises included interviews conducted with real playing (i.e., colleagues who proposed something they would like to change, in order to make the practice more realistic), as well as role playing (i.e., using cases that are built on the context of T1D). Furthermore, real-patient interviews were overseen by the psychologist, both directly and through visualization of video recordings, and feedback was provided. Finally, all researchers read the MI book “Motivational interviewing: helping people change” (15). In addition, as a reminder, they were given a magnet on which the main tasks associated with MI are printed, to be put up in a visible place in their unit.

**Table 1:** Contents of the theoretical-practical MI workshop.

*Spirit of MI*	Openness to a new model of thinking and doing with respect to patients. Developing active curiosity to understanding the patient’s perspectives, seeking to evoke the motivation for change in the patient him/herself. The spirit of the MI is based on four principles: collaboration, acceptance, evoking and compassion.
*Acquiring patient-centered counseling skills (OARS: open questions, affirmation, reflection, summary)*	Acquiring skills in the use of patient-centered communication. The key tools of MI are developed: empathy, open questions, affirmation, reflections for feedback, and summaries to take stock.
*Recognizing and reinforcing talk of change*	Developing the ability to recognize patients’ talk of change when it appears naturally in the context of ambivalence. Paying attention to expressions indicating desire, possibility, motives or need, and which predict the commitment to change.
*Eliciting and strengthening talk of change*	Helping professionals to strengthen talk of change once it has been recognized. The aim is to evoke talk of change deliberately instead of waiting for it to occur naturally.
*Rolling with resistance*	Learning to avoid resistance and responding to it appropriately if it appears. Practitioners should adjust to resistance rather than oppose it, through strategies of simple and complex reflections.
*Developing a change plan*	Developing the skill to attempt the transition from change talk to the development of an action plan to achieve it. The usual process is to offer a progressive summary of the change talk provided by the patient (desire, ability, motives and need) and then pose an open question whose essence is “What are you going to do now?”
*Consolidating patients’ commitment*	Once the plan is established, the crucial step is to maintain the patient’s commitment. The skills in this stage are the same as the ones developed in the other stages, since the fundamental thing is to listen to and support the patient’s language of commitment, understood as language that entails a decision once the action plan has been designed.
*Combining MI effectively with other tools*	MI is not the only tool in a professional’s repertoire. Patients who are prepared for the change should not be interviewed with MI, as it might be frustrating. It is part of the professionals’ task to recognize when to apply MI.

To avoid skill erosion and further improve MI performance, regular coaching and feedback-based, post-training sessions were held at baseline and are repeated at 2- to 3-month intervals during the intervention trial, to adhere to evidence-based practices (31). Individual reading of the book “Motivational Interviewing in Diabetes: facilitating self-care” (32) will complement the post-training sessions.

### Control Group

Patients randomly assigned to the control group will receive their four follow-up visits with standard medical care. The visits will be held with the same health practitioners, at the same intervals as in the intervention group, and will last a similar length of time. At each of the visits the HbA1c will be recorded, and before the first visit and 4 months after the fourth visit controls will complete the questionnaires in the same way as the intervention group.

### Interventions: Concomitant Care

Participants will continue with their usual follow-up routine for diabetes care. They are not asked for anything additional.

### Patient and Public Involvement

The results of the study will be presented to the scientific community at scientific meetings and in a research article. The results will also be disseminated in the local press and on the web page of the research group. Patients will also be informed of the results at the diabetes unit, once the trial has finished.

### Measures

The main variable of the study is adherence to self-care, measured through a validated questionnaire, the Diabetes Self-Care Inventory-Revised version (SCI-R). The secondary variables are: HbA1c, self-efficacy (EAG), health-related Quality of Life (ViDa1), satisfaction with the doctor-patient relationship, motivation for self-care, satisfaction with self-care, degree of fulfilment of a personal goal initially proposed by the participant, and a series of sociodemographic and clinical variables listed below. The time intervals for the measurements are shown in **Table 2**.

**Table 2:** Time points of measurements.

Variable	Appointment 1 baseline	Appointment 2 4m	Appointment 3 8m	Appointment 4 12m	Appointment 5 16m Post-intervention
Primary outcome measure					
Adherence to self-care (SCI-R)	X				X
Secondary outcome measures					
*HbA1c*	X	X	X	X	X
Self-efficacy (GSE)	X				X
Health-related quality of life (ViDa1)	X				X
Motivation for self-care	X				X
Satisfaction with self-care	X				X
Satisfaction with the doctor-patient relationship					X
Personal goal of diabetes care	X				X
Sociodemographic data	X				X
Clinical data	X				X

#### Adherence to Self-Care Behaviors

The validated Spanish version of the SCI-R (33) will be used. This inventory consists of 15 items referring to self-care behaviors in the treatment of diabetes, which are scored on a Likert scale ranging from (1 = “never” to 5 = “always”). The scores are converted with a formula, and the responses range from 0 to 100; higher scores indicate higher levels of self-care.

#### Structured Self-Administered Data Collection Sheet

This data sheet is designed specifically for the study and it covers the following sociodemographic and clinical variables: sex, age, level of education (unschooled, primary, secondary, and university studies), employment status, living arrangements, duration of disease, type of drug treatment, treatment with psychoactive drugs, carbohydrate count, level of training in diabetes, diabetes chronic complications, and the limitation they represented for participants’ daily lives, number of hypoglycemia episodes per week, number of daily capillary glucose readings, and previous history of acute complications (admissions for severe hyperglycemia or hypoglycemia). Medical variables are confirmed *via* the patients’ medical history.

#### HbA1c

Glycated hemoglobin (HbA1c) (measured by HPLC or by point-of-care Alere Afinion AS100, standardized against DCCT/NGSP) is the standard measure for evaluating glycemic control over the last 2–3 months. The ADA recommendations suggest a general target level for HbA1c of less than 7% to reduce risk of diabetic complications (11).

#### Self-Efficacy

The General Self-Efficacy Scale (34) is administered in its validated Spanish version (35). This scale measures respondents’ expectations regarding their ability to cope adequately with a problematic situation. Responses are recorded on a Likert scale (1 = “not at all” to 5 = “totally”) and the score range is 1–50. Higher scores indicate a higher perception of self-efficacy.

#### Health-Related Quality of Life

Health-related quality of life is measured with the ViDa1 (36), which contains 34 items grouped in four dimensions: interference of diabetes in daily life, self-care, well-being, and worry about the disease. The response format is a Likert scale ranging from (1 = “strongly disagree” to 5 = “strongly agree”). A total score is obtained for each subscale, with a higher value indicating a higher level of the respective aspect.

#### Motivation for Self-Care

Motivation for self-care is measured on a Likert scale of 1–10, where 10 is the maximum score. This item is formulated specifically for this study.

#### Satisfaction With Self-Care

Satisfaction with self-care is measured with a Likert scale of 1–10, where 10 is the maximum score. This item is formulated specifically for this study.

#### Doctor-Patient Relationship Satisfaction

Satisfaction with the doctor-patient relationship is measured with a Likert scale of 1–10, where 10 is the maximum score. This item is formulated specifically for this study.

#### Personal Goal

Patients set themselves a health-related goal which they aim to achieve through their participation in the trial. The degree of achievement is measured with a Likert scale (1–10).

#### Intervention Fidelity Assessment

To evaluate the application of MI by the practitioners, video recordings of 25 patients were carried out at the first visit. At the fourth visit, all patient interviews will be recorded, and a random sample of 25 will be evaluated. A mobile phone with a tripod and an external memory card are used to make the video recordings. The recordings will be evaluated by two external observers using the Motivational Interviewing Rating Scale (MIAS/EVEM 2.0) (37). This scale has been previously validated for the application of MI in primary care; applied to several clinical sessions, it discriminates between the sessions in which the method has been used (and establishes to what degree) and the ones in which it has not been used. The raters will not know *a priori* to which group the patients in the recordings have been assigned.

### Analysis

Based on the number of patients followed in the clinic and the percentage with an HbA1c above 8%, we estimated that each of the three clinical researchers involved in the trial would be able to recruit around 20 patients in 4 months. With an expected sample size of 60 participants, we would be able to detect inter-group differences of 12.8 points on the SCI-R questionnaire and of 0.85% in HbA1c, with a power of 90% and a two-sided alpha of.05 [assuming an SD of 15 points in the SCI -R and 1% in HbA1c in line with previous studies (5)]. The equivalent detectable differences with a power of 0.8 would be 11 points and 0.74% respectively (calculated with http://hedwig.mgh.harvard.edu/sample_size/js/js_parallel_quant.html). This was an approximate estimation assuming the normality of the variable.

In order to retain most of the patients during follow-up, if someone misses an appointment, the patient is contacted and the visit is re-scheduled. Specific efforts will be made for the final evaluation visit. If a physical appointment cannot be achieved, the questionnaires will be sent and completed by e-mail or, if this is not feasible, the patient with be interviewed by phone. We expect the loss to follow-up to be below 10%.

Intention to treat analysis will be performed: i.e., all randomized participants who have attended at least one intervention (or control) session will be included in the analyses. The main analysis for primary and secondary outcomes, except HbA1c, will be a generalized linear model (GLM) including the intervention arm, the baseline score of the dependent variable and other variables imbalanced at baseline as covariates. The dependent variable will be the post-intervention score. The GLM does not need a normal distribution in the dependent variable (38), although the distribution of the variables will be analyzed using de Shapiro- Wilk test to perform an adequate descriptive analysis. The choice of the link function will depend on the distribution of the variable.

For the HbA1c, a GLM for multilevel analysis will be used including the intervention arm, baseline HbA1c and other imbalanced variables in the baseline as covariates. First-level are those corresponding to each measurement along follow-up (repeated time measurements); the second level includes patients’ variables. The model will also include an interaction term between the intervention-arm and time (follow-up), allowing for differences in the intervention effect between follow-up assessments (39). The effect that identifies the intervention arm is considered fixed, whereas the intercept is considered random. An autoregressive [AR (1)] covariance structure will be used in the model.

Other variables will be added as confounders in the model, as secondary sensitivity analyses. We will first fit separate models including each confounder, one at a time. Those variables whose inclusion in the model changes the estimates’ treatment effect by at least 10% will be considered as confounders. As suggested in the CONSORT statement, decisions about confounders will not be based on P value  (40).

In the case of missing values, these will be accommodated with multiple imputation procedure in Stata 15.0 software (Stata Corporation) (41). This procedure saves cases for the analysis and can be considered an intention-to-treat analysis. Analysis under multiple imputations is valid for randomly missed data (42). A threshold of.05 will be used to define the statistical significance of those tests.

For the MIAS/EVEM 2.0 scale, the Intraclass Correlation Coefficient (ICC) will be used.

## Discussion

This study protocol presents the design of a randomized controlled clinical trial aimed to evaluate the impact of an MI intervention on the self-care behaviors of people with T1D. We expect the MI-based intervention to improve the doctor-patient relationship and to increase the motivation for self-care, and with it, self-care behaviors. We also expect improvements in glycemic control and increases in self-efficacy and health-related quality of life.

There is evidence that MI can improve self-care in T2D (20, 21). In T1D, to date, however, its application has focused on adolescents, in whom some studies have shown a statistically significant improvement in glycemic control (21–24, 29, 43) but others have not (27). At present, there are no published data on the effect of MI in adults with T1D.

Increasing adherence to self-care is a challenge in a chronic disease such as T1D. An MI-based intervention to replace the traditional interview may improve glycemic control and thus prevent chronic complications in the future. In this study, we aim to evaluate the effect of MI on self-care and HbA1c.

We acknowledge that this study has some limitations. MI is a complex tool to learn; it takes time to manage it correctly, practitioners may not be experts in its use and learned skills tend to wear off. In order to reduce training erosion, regular coaching and feedback will be provided throughout the trial. On the other hand, the same practitioner will apply MI to some patients and the traditional interview to the others (control group), which carries a certain risk of contamination between the study groups. To minimize contamination, participants seen by the same practitioner on the same day were assigned to the same treatment group, which could, of course, lead to some imbalance between the sizes of the groups. To monitor if MI is indeed applied in the intervention group and not in the control group, the sessions will be blindly evaluated at the first and last visits of the study. Finally, not all patients are comfortable with this method of interviewing, a situation that may hinder its application. Regarding outcomes, self-care, the primary outcome, comprises an array of behaviors, which might not accurately reflect the success of MI if change is focused on a single behavior, selected by the patient. The assessment of the achievement of self-defined goals should, at least partially, account for this drawback. Furthermore, some concepts will be examined on an exploratory basis using only a single-item and not a psychometrically tested measure, so these findings should be interpreted as descriptive and with caution. Finally, the sample size of this trial will be able to detect relatively large, clinically relevant changes in the primary and some of the secondary endpoints. However, it might be underpowered to detect smaller, albeit still relevant effect sizes. The low number of healthcare professionals included in the trial, which act as randomization clusters, does not allow the effect associated with the health professionals to be included in the analysis. This could potentially lead to bias, since the variance associated with the healthcare professionals cannot be estimated.

## Confidentiality

All study-related information will be stored securely at the study site. All participant information will be stored in locked file cabinets in areas with limited access. All reports, data collection, process, and administrative forms will be identified by a coded ID [identification] number only to maintain participant confidentiality. All records that contain names or other personal identifiers, such as locator forms and informed consent forms, will be stored separately from study records identified by code number. All local databases will be secured with password-protected access systems. Forms, lists, logbooks, appointment books, and any other listings that link participant ID numbers to other identifying information will be stored in a separate, locked file in an area with limited access or in secure, password-protected electronic files.

## Model Consent

The informed consent model used in this study is the one established by the local Ethics Committee.

## Trial Sponsor

AW. Address: Endocrinology and Nutrition Dept. Complejo Hospitalario Universitario Insular Materno-Infantil Gran Canaria, Av. Marítima s/n. 35016, Las Palmas de Gran Canaria, Spain. Tel: +34 928453431. FAX: +34 928442586.

## Protocol Amendments

Any modifications to the protocol which may impact on the conduct of the study, potential benefit of the patient or may affect patient safety, including changes of study objectives, study design, patient population, sample sizes, study procedures, or significant administrative aspects will require a formal amendment to the protocol. Such amendment will be agreed upon approved by the Ethics Committee of Las Palmas prior to implementation.

## Data Access

All investigators will have access to the trial data sets. Project data sets will be housed locally and will be password protected. Access to cleaned, anonimized data sets will be available to external investigators upon request. The present manuscript does not include data.

## Data Collection Plan: Retention

Participants may withdraw from the study for any reason at any time. In the informed consent model, a section is included for the participant to withdraw their participation if they deem it appropriate. Efforts will be made to minimize drop-outs, as described above.

## Ethics Statement

The study was reviewed and approved by CEIm Provincial Hospital Universitario de Gran Canaria Dr. Negrin (HUGCDN). Barranco de la Ballena s/n. Hospital Universitario de Gran Canaria Dr. Negrín, Edificio de Investigación, Planta principal. 35019 Las Palmas de Gran Canaria (Las Palmas) Phone: (+34)928 450971 – 928 449071– 928 449286 ceimprovlpa.scs@gobiernodecanarias.org. The patients/participants provided their written informed consent to participate in this study.

## Author Contributions

DA-M participated in the design of the study, provided MI training for practitioners, contacted the participants by phone, performed the interviews to complete the questionnaires, and wrote the first draft of this article. MB participated in the study design, received MI training, recruited patients, applied MI to the participants, and contributed to the writing of this article. MP received MI training, recruited patients, and applied MI to the participants. MA-G received MI training and conducted interviews to complete the questionnaires. AW participated in the design of the study, received MI training, recruited patients, applied the MI to the participants, and contributed to the writing of this article. YR-F designed the statistical plan. All authors contributed to the article and approved the submitted version.

## Funding

This work is supported by and unrestricted grant from the Menarini Group, Spain, as well as by a competitive, peer-reviewed grant from Fundación Mapfre Guanarteme [PI 2019], Las Palmas de Gran Canaria, Spain. The funders have had no role in the design of the trial and will have no role in the collection, analysis or interpretation of the data, nor in the preparation or publication of the manuscripts related to this trial.

## Conflict of Interest

The authors declare that the research was conducted in the absence of any commercial or financial relationships that could be construed as a potential conflict of interest.

## Abbreviations

MI, motivational interviewing; MINT, Motivational Interviewing Network of Trainers; T1D, type 1 diabetes; T2D, type 2 diabetes.
